# A System of Surgery

**Published:** 1897-01

**Authors:** 


### Book Reviews.

A SYSTEM OF SURGERY. In Contributions by Twenty-five English Authors. Edited by FREDERICK TREVES, F. R. C. S., Surgeon to, and Lecturer on, Surgery at the London Hospital ; Examiner in Surgery at the University of Cambridge. Volume II. In one 8vo volume of 1,120 pages, with two colored plates and 487 illustrations. Philadelphia and New York : Lea Brothers & Co. 1896.

This, the second volume of Treves's Surgery, is in every way a fitting companion to the first volume, which was given to the profession several months ago. The subjects comprise the surgical diseases and injuries of the muscular and osseous systems, the head and neck, chest and abdomen and the genito-urinary organs, male and female.

The chapters upon the injuries and diseases of the head, by Henry Percy Dean, are excellent. The subject matter, which shows large information and experience, is ably supplemented with illustrative figures, many of which are new. This is particularly true of that portion which deals with the various forms of fracture of the skull. The section upon abscess of the brain is a further illustration of the author's ability to put a great deal of information in few words. Another admirable but short chapter is that by Herbert W. Page upon Concussion of the spine. The author's remarks upon so-called "railway spine" deserve to be read by every practising physician and surgeon. We regret that space prevents at least a good abstract of this chapter, for in it can be found much wholesome advice and information upon a subject concerning which there seems to be many erroneous ideas, and certainly one will be taught through larger knowledge to get a fairer and juster view of the patient's side of the affair.

The chapter dealing with the surgical conditions occurring within the abdomen is perhaps the best in the volume. Here, in about 100 pages, the editor has given us the results of a large experience. The subject matter is arranged in a most orderly and systematic manner, which makes the information of easy access to the reader. From the operative point of view, the author will, in most if not in all instances, be found properly radical and conservative. For instance, he says: "An incision in perityphlitis is very seldom called for before the fifth day," and further on cautions against reckless and unnecessary incisions made during the first two or three days of the attack. Regarding relapsing perityphlitis, the best treatment for warding off a later attack is attention to the diet, the bowels and general mode of living of the patient. He even declares that a plate of false teeth has cured more than one case of relapsing perityphlitis. The section upon intestinal obstruction is a most instructive one. The chapter upon hernia is also by the editor, and is a condensed but complete presentation of this ever-interesting and important subject. We are surprised to see that the operation of McBurney is still mentioned in terms which would indicate that he (McBurney) still believed in and practised the operation, notwithstanding its public repudiation by him over two years ago.

Diseases of the breast receive consideration by W. Watson Cheyne, who contributes a thoroughly good article upon this subject. His views and therapeutic suggestions in carcinoma are thoroughly modern and can be safely followed to the advantage of the patient.

Henry Morris contributes two chapters upon genito-urinary diseases. They are characteristic of the author's work, which has such an international reputation as to need no further comment.

The publishers have kept up their general standard of excellence and given to the profession a work most acceptable in every way.

